# P-1672. Novel Software and Web Application to Facilitate and Simplify Antibiotic Stewardship to Improve Antibiotic Days of Therapy (DOT) and Cost Savings with Evidenced-Based Algorithms in Long Term and Post Acute Care Facilities (LTPAC)

**DOI:** 10.1093/ofid/ofae631.1838

**Published:** 2025-01-29

**Authors:** Steve J Williams

**Affiliations:** IDCare, basking ridge, New Jersey

## Abstract

**Background:**

According to the Centers for Disease Control (CDC), 4.1 million Americans are admitted to reside in LTPAC during a year, 70% of those receive antibiotics, and only 25% are used correctly prompting a 2017 mandate for Antimicrobial Stewardship by the Center for Medicare and Medicaid Services (CMS). Most facility programs miss many of the seven core elements as outlined by CDC (Fig 1) lacking the expertise and oversight to improve antibiotic use, decrease days of therapy, and reduce resistant organisms and adverse outcomes.

**Figure 1.**
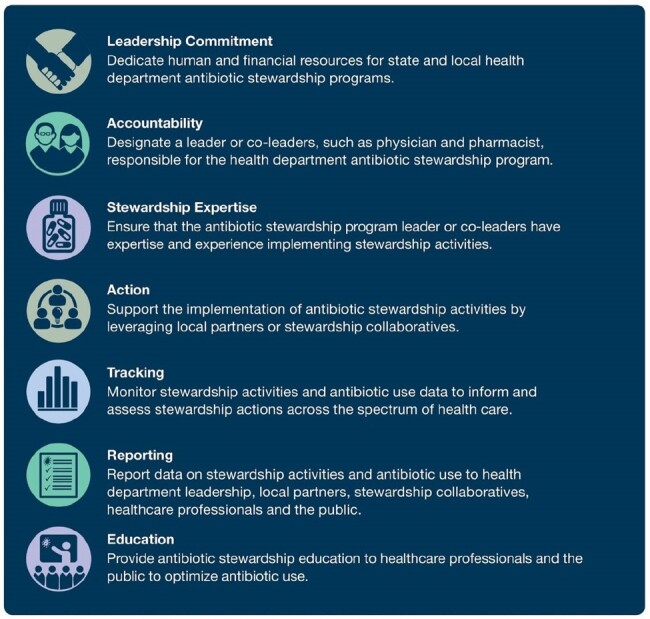
Core Elements of LTPAC Antibiotic Stewardship Programs

**Methods:**

A stewardship software system was developed for LTPAC at www.idsteward.com. Six facilities were evaluated for 2023. Entries were categorized as facility-acquired infections and other-acquired infections. Newly diagnosed facility infections were primarily analyzed and subject to 2020 updated McGeer screening criteria. Entries were reviewed for length of therapy, diagnosis, and substitutions (Fig 2), and released back as a reviewed “stewardship recommendation(s)” in real-time, indicating neutral, agree, disagree, modify, or disagree with proposed therapy. Cost was estimated as the mean for each antibiotic through Lexidrug. DOT and costs were calculated for both the provider and the “Steward”. Providers of facilities were compared to the Steward and received feedback in real-time per facility discretion as well as quarterly.

**Figure 2.**
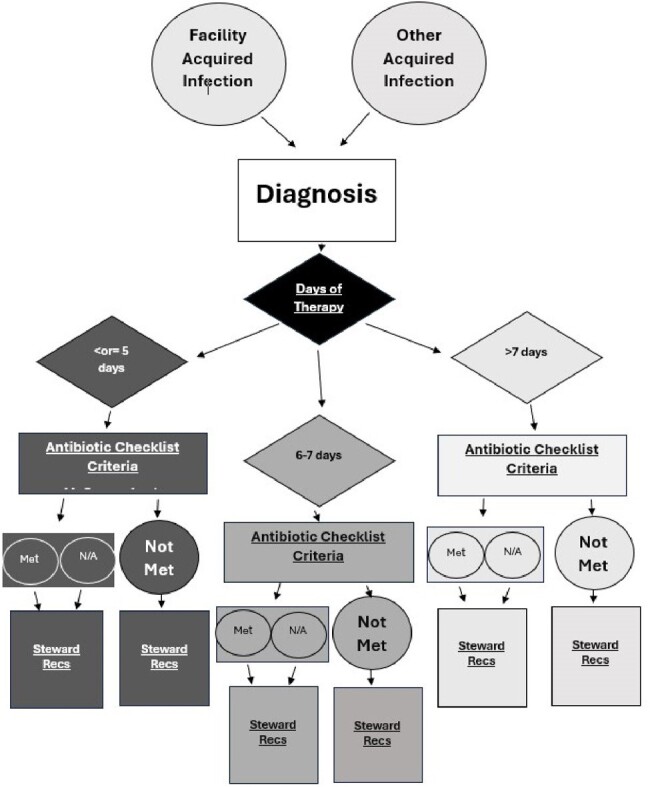
Sample algorithm

**Results:**

Six Facilities were evaluated via the software for 2023. A total of 14320 days of therapy were observed by the providers (Fig 3). A total of 13666 antibiotic days were realized by the steward with a total 654 DOT saved, or a 4.6% decrease (Fig 4). Cost savings for those DOT saved and antibiotic substitutions were estimated at $34692 total ($5782 average).

**Figure 3.**
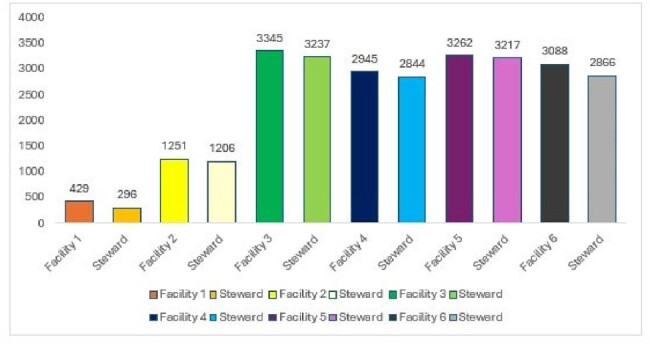
Days of Therapy Analysis

**Conclusion:**

Antimicrobial stewardship in LTPAC poses many obstacles given their challenges with staffing, lack of resources to antibiotic expertise, and provider oversight and education. Antibiotic usage by a facility can be supervised with a software-based automated algorithm that can recommend real-time changes to antibiotic length of therapy based on standardized screening criteria such as McGeer, resulting in saved DOT and cost.

**Figure 4.**
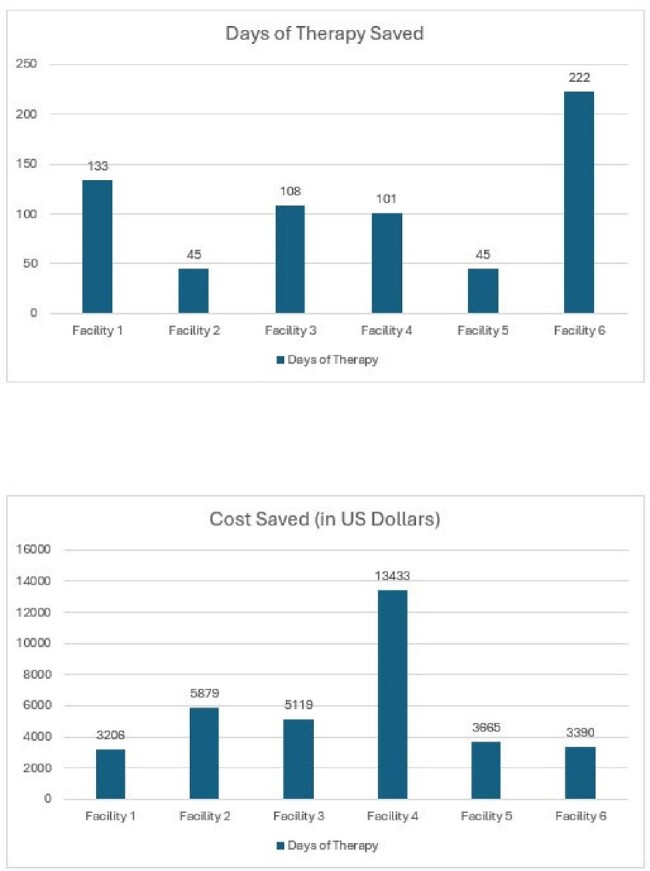
Days of Therapy Saved and Cost Saved

**Disclosures:**

**All Authors**: No reported disclosures

